# Neutrophil‐to‐lymphocyte ratio and red blood cell distribution width as predictors of microalbuminuria in type 2 diabetes

**DOI:** 10.1002/jcla.23259

**Published:** 2020-02-25

**Authors:** Tikva Assulyn, Rola Khamisy‐Farah, William Nseir, Amir Bashkin, Raymond Farah

**Affiliations:** ^1^ Galilee Medical Center Nahariya Israel; ^2^ Azrieli Faculty of Medicine Bar‐Ilan University Safed Israel; ^3^ Clalit Health Services Akko Israel; ^4^ Department of Internal Medicine A Baruch Padeh Medical Center Poriya Israel; ^5^ Department of Internal Medicine B Ziv Medical Center Safed Israel

**Keywords:** diabetic nephropathy, inflammatory markers, microalbuminuria, neutrophil‐to‐lymphocyte ratio, type 2 DM

## Abstract

**Background and aim:**

Chronic inflammation has an important role in the development and progression of type 2 diabetes through immunologic inflammatory mechanisms. Simple new inexpensive inflammatory markers may contribute to the detection of microalbuminuria. Aim of our study is to evaluate the predictive value of neutrophil‐to‐lymphocyte ratio (NLR), mean platelet volume (MPV), and red blood cell distribution width (RDW) for microalbuminuria in type 2 diabetic patients for possible application as prognostic factors for the prediction of microalbuminuria and the progression of disease in patients with diabetes.

**Methods:**

A total of 168 patients with type 2 diabetes mellitus were classified into gender‐ and BMI‐matched three groups according to hemoglobin A1c and microalbuminuria: Group A: 53 patients with controlled diabetes, Group B: 57 patients with uncontrolled diabetes, both without microalbuminuria, and Group C: 58 patients with uncontrolled diabetes with microalbuminuria. Levels of NLR, MPV, and RDW between the study groups were examined and compared.

**Results:**

A significant difference in NLR was found between Group C and groups A and B (*P* < .001, *P* = .005, respectively). A statistically significant difference in RDW was found between groups B and C (*P* = .014). Receiver operating characteristic curve analysis of inflammatory markers and microalbuminuria prediction showed an area under curve (AUC) of 0.675 for NLR (CI 0.58‐0.76, *P* < .001) and 0.614 for RDW (CI 0.52‐0.70, *P* = .013). NLR value of 2.54 has 39.7% sensitivity, 78.8% specificity, and 45% positive predictive value (PPV). RDW value of 14.44 has 37.9% sensitivity, 76% specificity, and 41.5% PPV.

**Conclusions:**

Neutrophil‐to‐lymphocyte ratio and RDW have PPV for microalbuminuria in diabetic patients.

## 1 INTRODUCTION

Chronic inflammation has an important role in the development and progression of type 2 diabetes through immunologic inflammatory mechanisms. The neutrophil‐to‐lymphocyte ratio (NLR) is a new, simple, and inexpensive marker of subclinical inflammation^1^ and has been used recently as a systemic inflammatory marker in chronic diseases as well as a predictor of prognosis in cardiovascular diseases, malignancies, and metabolic syndrome 2, 3; in addition, NLR has been designated as a measure of systemic inflammation in different stages of chronic kidney disease (CKD), diabetic nephropathy, and the usefulness to predict adverse outcomes in medical and surgical conditions.^4^, ^5^, ^6^ However, the value of NLR in predicting diabetic nephropathy has not yet been elucidated.^6^ Leukocyte activation occurs during an inflammatory reaction. Leukocytes have a role in atherogenesis and thrombus formation. Elevated levels of inflammatory mediators in the circulation have been associated with the development of cardiovascular disease.^7^, ^8^ Other studies showed that elevated NLR in otherwise healthy subjects may be indicative of underlying impaired glucose metabolism, and moreover, NLR should be used as a marker of diabetic control level in addition to HbA1c in type 2 diabetic subjects.^9^ A retrospective Turkish study has demonstrated a positive correlation between high levels of NLR, HbA1c, serum creatinine, and systolic blood pressure and longer duration of diabetes, concluding that NLR is an efficient, cheaper, and readily available marker of inflammation, and it is known as an important predictor for the existence of microvascular complications in subjects with type 2 diabetes.^9^, ^10^

A symptom of nephropathy is albuminuria usually initiating with microalbuminuria, which was found to be a marker of vascular endothelial damage.^11^ Albuminuria is a well‐known predictor of poor renal outcomes in patients with type 2 diabetes and in essential hypertension,^12^ and as such, it is important to monitor and diagnose in order to treat early. Microalbuminuria is a microvascular complication of diabetes. Without intervention, diabetic patients with microalbuminuria typically progress to proteinuria and overt diabetic nephropathy. This progression occurs in both type 1 and type 2 diabetes.^13^ In addition, in type 2 diabetic patients, microalbuminuria is accompanied by elevated CRP, suggesting activation of inflammatory pathways in progression of renal and cardiovascular atherosclerotic disease.^14^

Mean platelet volume (MPV) is a machine‐calculated measurement of the average size of platelets found in blood and is typically included in blood tests as part of the CBC. Since average platelet size is larger when the body is producing increased numbers of platelets, the MPV test results can be used to make inferences about platelet production in bone marrow or platelet destruction problems. Elevated MPV is an indicator of inflammation due to increased destruction to platelets.^15^ One study showed a significant positive relationship between microalbuminuria and MPV and also found that among type 2 diabetic patients, MPV is higher in those who have microvascular complications such as retinopathy or microalbuminuria.^16^

Red blood cell distribution width (RDW) is a measure of the range of variation of red blood cell (RBC) volume that is also included in blood tests as part of the CBC. Studies showed that RDW might be considered as an effective predictive index in the evaluation of diabetic nephropathy or diabetes‐associated complications.^17^, ^18^ Zhang et al 18 conducted a retrospective analysis which extended the previous finding that RDW is significantly associated with HbA1c in a large cohort of elderly European outpatients. Other study showed that RDW and RDW/MCV ratio were found associated with diabetic ketoacidosis (DKA) and valuable in predicting DKA.^19^ Since RDW is a simple and inexpensive parameter, it may be considered a potential, innovative biomarker for improving risk assessment of developing diabetes and for the prediction of diabetic complications.

Against this background, we aimed to conduct a study in order to examine the possible role of NLR, MPV, and RDW as fast and cheaper inflammatory markers in predicting microalbuminuria in type 2 diabetic patients, especially in hospitals and clinics that are unable to perform microalbumin‐creatine ratio. Another reason supporting the use of these markers, is pevention some misinterpretation of urine collection, extra cost and time of urine collection analysis.

## 2 MATERIALS AND METHODS

### 2.1 Design, setting, and sample

Three hundred and twenty medical records of patients with diabetes from the outpatient clinics at Ziv Medical Center, Safed, and Galilee Medical Center, Nahariya, Israel, during the years 2014‐2017 were reviewed and examined. The chart reviews were performed by one resident. There was no inadvertent bias during the review process. Of these, the data from 168 patients with type 2 diabetes mellitus (DM) met the inclusion criteria of the study. We included patients aged ≥40 years old who had type 2 DM for at least 4 years. We excluded patients with recent diagnosis of acute infection or inflammation, leucocytosis, leukopenia, severe anemia, chronic infection, chronic systemic inflammatory disease, medications affecting the number of leukocytes, uncontrolled hypertension, and secondary hypertension, any chronic kidney injury, end stage kidney disease, hepatic failure, and/or manifest active heart disease such as cardiac failure, acute coronary syndrome, arrhythmia, and cardiac valve disease.

Patients were classified into three groups according to glycated hemoglobin A1c and microalbuminuria: Group A, patients with controlled diabetes; Group B, patients with uncontrolled diabetes (both groups without microalbuminuria); and Group C, patients with uncontrolled diabetes with microalbuminuria. A sample size calculation was performed to determine the number of subjects that would be needed to answer the study questions. The study was reviewed and approved by Ethics (Helsinki) Committees at both Ziv Medical Center and Galilee Medical Center.

### 2.2 Measurements

We recorded the demographic, clinical, and laboratory data from patient file records regarding plasma glucose, HbA1c, creatinine, albumin, total cholesterol, triglycerides (TG), high‐density lipoprotein (HDL), and low‐density lipoprotein (LDL) levels in the venous blood samples obtained in the morning after 8‐h fasting. Body mass index (BMI) (kg/m^2^), body surface area (m^2^) and blood pressure values were collected from patient files using the formulas BMI = weight (kg)/height (m)^2^ and BSA (m^2^) = 0.007184 × Height (cm)^0.725^ × Weight (kg)^0.425^. Complete blood counts were analyzed in the hematology unit with a Beckman‐Coulter Gen‐S system device (Beckman‐Coulter Inc.). MPV and RDW levels were gathered from patients' complete blood count, and NLR was calculated as the ratio of absolute number of neutrophil and lymphocyte counts. For each patient, two consecutive blood tests were evaluated and checked for consistency of the parameters listed above, to exclude irregularities. For the exclusion of a mild disease, all blood counts with WBC above 9000/µL were not included. All the measurements collected including CBC, electrolytes, NLR, RDW, MPV, and urine albumin were performed at the same time, and within 3 weeks of the last visit, all the groups their kidney function tests were within the normal range and suffering only from diabetes.

### 2.3 Definitions

Uncontrolled DM was considered when HbA1c >7%.^20^ Microalbuminuria was examined from a morning spot urine, defined as albumin‐to‐creatinine ratio of >2.5 mg/mmol for men and >3.5 mg/mmol for women, or 30‐299 mg/24 h which corresponds to 20‐200 µg\min both sexes.^12^

### 2.4 Data analysis

Continuous variables are defined as mean ± standard deviation, and categorical variables are given as frequencies and percentages. We used one‐way analysis of variance (ANOVA) tests to compare among the three study groups. Statistical significance and power required were 5% and 80%, respectively. A post hoc with Tukey's multiple comparisons analysis was performed in parameters in which a significant difference was found. Correlations were assessed using Pearson's test. Receiver operating characteristic (ROC) curve analysis was used to determine the optimum cutoff levels of inflammation markers NLR and RDW to predict microalbuminuria. Statistical analysis was performed using Statistical Package for Social Sciences (SPSS) version 19.0 (SPSS Inc.). Any *P* value <.05 was considered statistically significant.

## 3 RESULTS

### 3.1 Demographic, clinic, and laboratory features of the study groups

Significant differences were found between groups A and C in the age variable (*P* = .02) and in years of diabetes (*P* = .01). TG/HDL ratio was different in patients with controlled diabetes (group A) compared to the other two groups (groups A and B, *P* = .02; groups A and C *P* = .01). Serum creatinine was higher and different in group C compared to the other two study groups (*P* < .001). Patients were categorized into groups using HbA1c and microalbumin‐to‐creatinine ratio levels and so they were significantly different in these parameters, as expected. No differences were found between the groups in gender, blood pressure (by mean arterial pressure), BMI, albumin, or LDL (Table 1).

**Table 1:** Demographic, clinic, and laboratory features of the study groups

	Group A: Controlled DM without microalbuminuria	Group B: Uncontrolled DM without microalbuminuria	Group C: DM with microalbuminuria	*P* value
	Mean ± Std. Dev	Mean ± Std. Dev	Mean ± Std. Dev	
Age (y)	64 ± 11	61 ± 10	67 ± 10	.02
Years of diabetes	10 ± 6	14 ± 8	15 ± 7	.01
Male/female (N)	24/29	27/30	33/25	
BMI (kg/m^2^)	28.81 ± 5.64	29.06 ± 4.73	29.80 ± 4.97	.56
HbA1c (%)	6.30 ± 0.46	8.43 ± 1.08	8.54 ± 1.66	.00
MAP (mm Hg)	96 ± 8	96 ± 9	96 ± 9	.8
Albumin (g/dL)	4.28 ± 0.34	4.24 ± 0.33	4.21 ± 0.34	.60
Serum creatinine (mg/dL)	0.80 ± 0.19	0.82 ± 0.24	1.11 ± 0.47	.00
TG/HDL ratio	2.87 ± 1.68	4.36 ± 3.12	4.61 ± 3.46	.04
LDL‐C (mg/dL)	87.95 ± 28.43	83.53 ± 36.31	85.59 ± 31.13	.77
microalbumin:creatinine ratio (mcg/mg)	6.15 ± 5.77	8.37 ± 6.95	228.79 ± 327.56	.00
NLR	1.90 ± 0.65	2.06 ± 0.83	2.60 ± 1.19	<.001
MPV (fL)	9.96 ± 1.53	10.06 ± 1.53	10.09 ± 1.44	.88
RDW (%)	14.04 ± 0.98	13.85 ± 1.17	14.48 ± 1.34	.016

Abbreviations: BMI, body mass index; DM, diabetes mellitus; HbA1c, glycated hemoglobin; LDL‐C, low‐density lipoprotein cholesterol level; MAP, mean arterial pressure; MPV, mean platelet volume; N, number of patients included per group; NLR, neutrophil‐to‐lymphocyte ratio; RDW, red blood cell distribution width; Std. Dev, standard deviation; TG/HDL ratio, triglyceride (TG) to high‐density lipoprotein (HDL).

### 3.2 Study groups differences in inflammatory markers

Table 1 shows significant differences in NLR and RDW in the three groups (*P* < .001; *P* = .016), respectively. No significant difference was found in MPV among the groups (*P* = .889).

**Figure 1:**
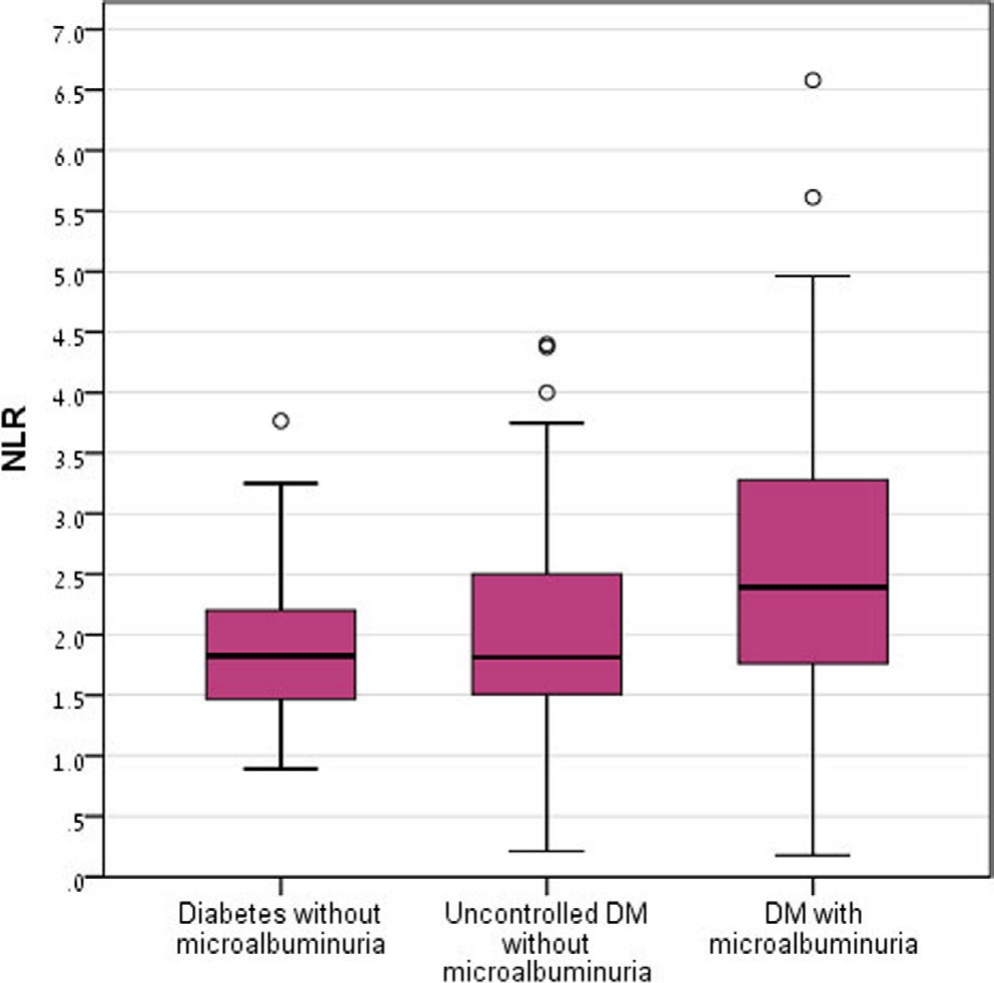
Neutrophil‐to‐lymphocyte ratio distribution in the study groups. DM, diabetes mellitus

**Figure 2:**
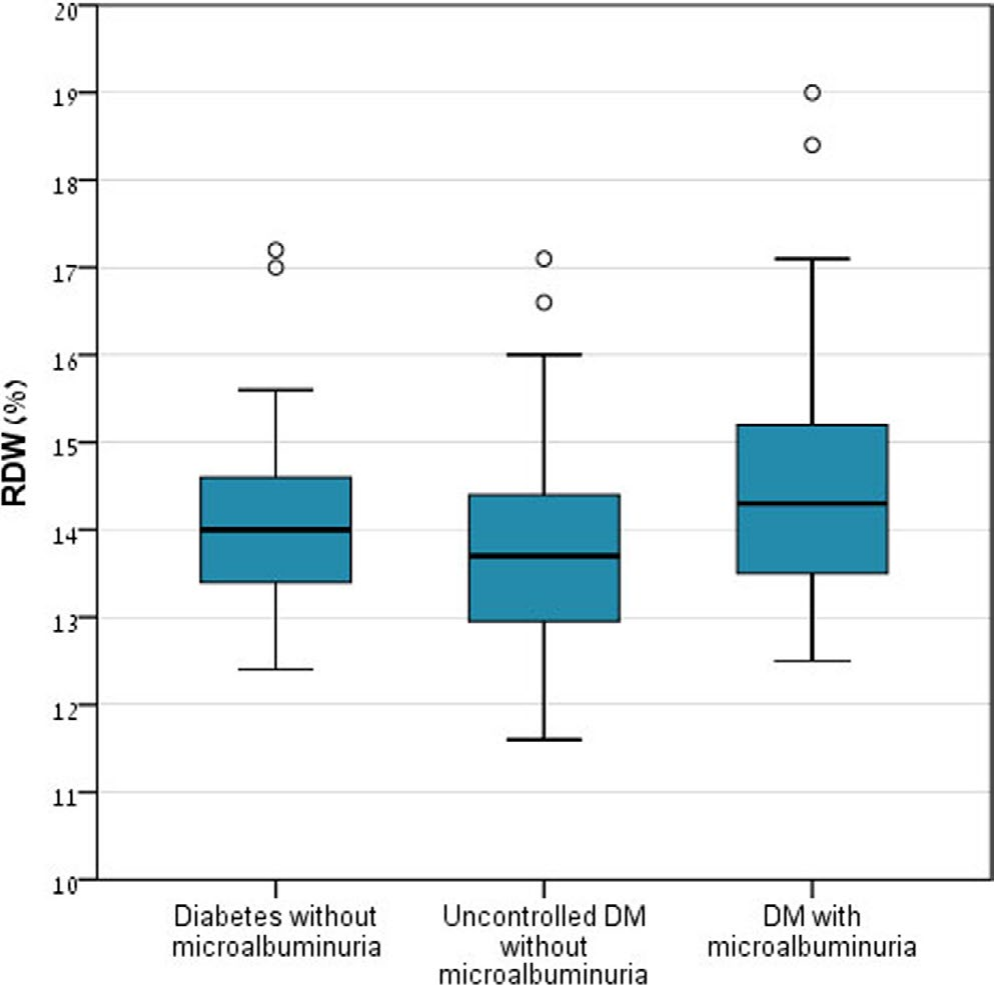
Red blood cell distribution width distribution in the study groups. DM, diabetes mellitus

A post hoc analysis for examination of the differences in NLR and RDW as performed can be seen in Table 2. A statistically significant difference in NLR was found between groups A and C (*P* value <.001) and between groups B and C (*P* value = .005). A statistically significant difference in RDW was found between groups B and C (*P* value = .014). No significant difference was found between groups A and B. Graphical representation of NLR distribution is shown by box plot in Figure 1 and of RDW distribution in Figure 2.

**Table 2:** Post hoc analysis for examination of the differences in NLR and RDW between the study groups

Dependent variable	(I) GROUP	(J) GROUP	Mean difference (I‐J)	*P* value
NLR	A: Controlled DM without microalbuminuria	B: Uncontrolled DM without microalbuminuria	−0.154	.658
		C: DM with microalbuminuria	−0.702	**<.001**
	B: Uncontrolled DM without microalbuminuria	A: Controlled DM without microalbuminuria	0.154	.658
		C: DM with microalbuminuria	−0.548	**.005**
	C: DM with microalbuminuria	A: Controlled DM without microalbuminuria	0.702	**<.001**
		B: Uncontrolled DM without microalbuminuria	0.548	**.005**
RDW %	A: Controlled DM without microalbuminuria	B: Uncontrolled DM without microalbuminuria	0.193	.677
		C: DM with microalbuminuria	−0.435	.138
	B: Uncontrolled DM without microalbuminuria	A: Controlled DM without microalbuminuria	−0.193	.677
		C: DM with microalbuminuria	−0.6276	**.014**
	C: DM with microalbuminuria	A: Controlled DM without microalbuminuria	0.435	.138
		B: Uncontrolled DM without microalbuminuria	0.6276	**.014**

Abbreviations: NLR, neutrophil‐to‐lymphocyte ratio; RDW, red blood cell distribution width.

Bold values indicate statistical significant.

### 3.3 Analysis of differences in the laboratory features of the study groups

**Figure 3:**
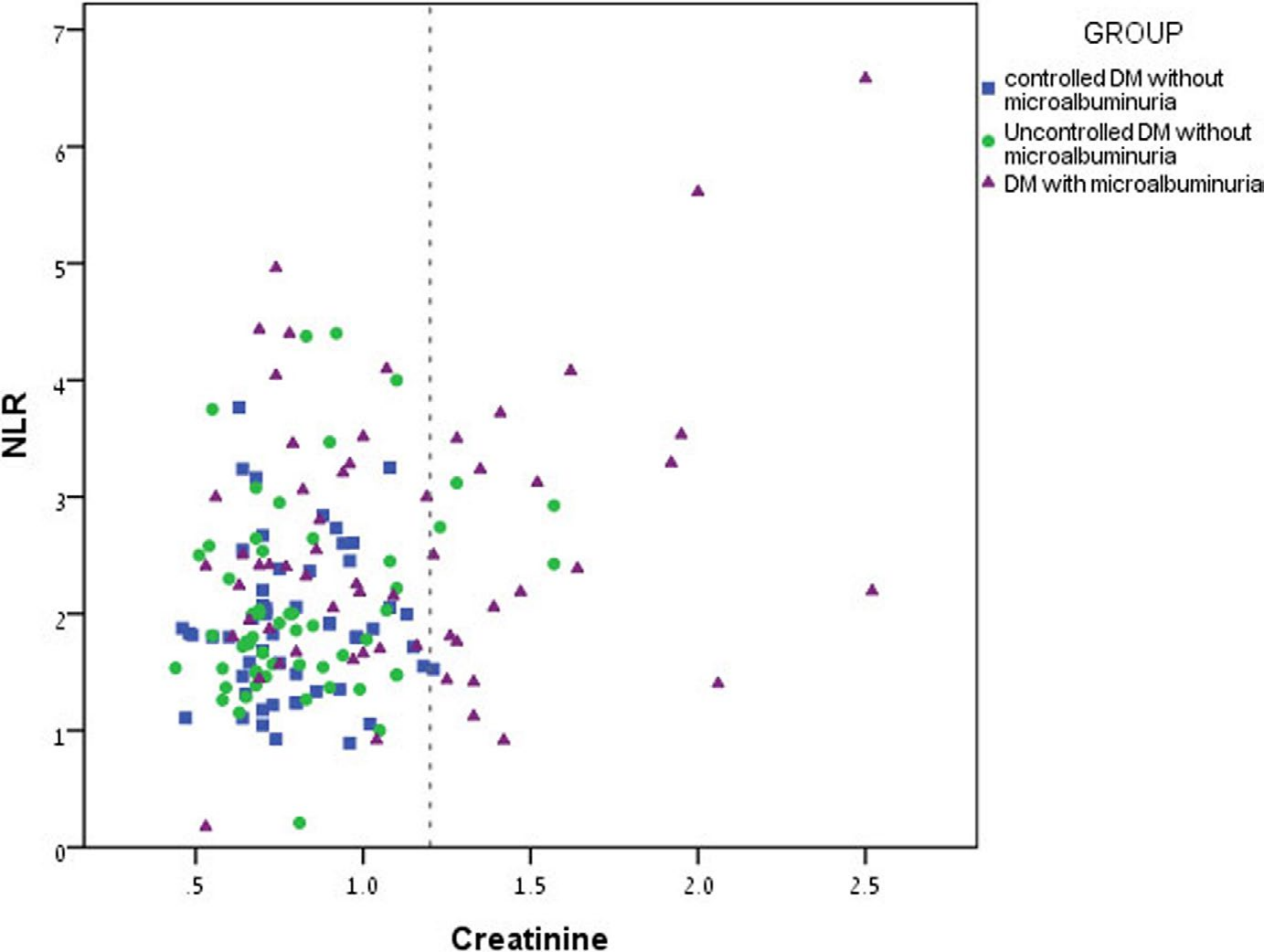
Scatter plot graphically presenting creatinine levels in the study groups: Group A presented in square shape, Group B presented in circle shape, and Group C presented in triangle shape. Note: creatinine units mg/dl

A scatter plot graphically presenting creatinine levels in the study groups is shown in Figure 3. Group C had a higher incidence of patients with creatinine level above the normal range (21 patients, 36.2% of the group), compared to the other two study groups (1.9% in group A, 7% in group B).

### 3.4 Correlations between the inflammatory markers and microalbuminuria

Univariate analysis (Pearson) found significant correlation between NLR and creatinine, HbA1c, and microalbuminuria, and NLR and RDW (Table 3).

**Table 3:** General Pearson's correlation analysis in parameters that were found to be significant

	Creatinine	HbA1c (%)	Microalbumin mcg/mg	RDW
NLR				
Pearson's correlation (*r*)	.300**	.183*	.214**	.174*
*P* value (2‐tailed)	**.000**	**.011**	**.003**	**.017**

Abbreviations: HbA1c, glycated hemoglobin; NLR, neutrophil‐to‐lymphocyte ratio; RDW, red blood cell distribution width.

*^,^ ** Means that a significant correlation was found between NLR and creatinine and microalbumin more than HbA1c and RDW. Bold values indicate statistical significant.

### 3.5 Receiver operating characteristic curve analysis for microalbuminuria prediction

**Figure 4:**
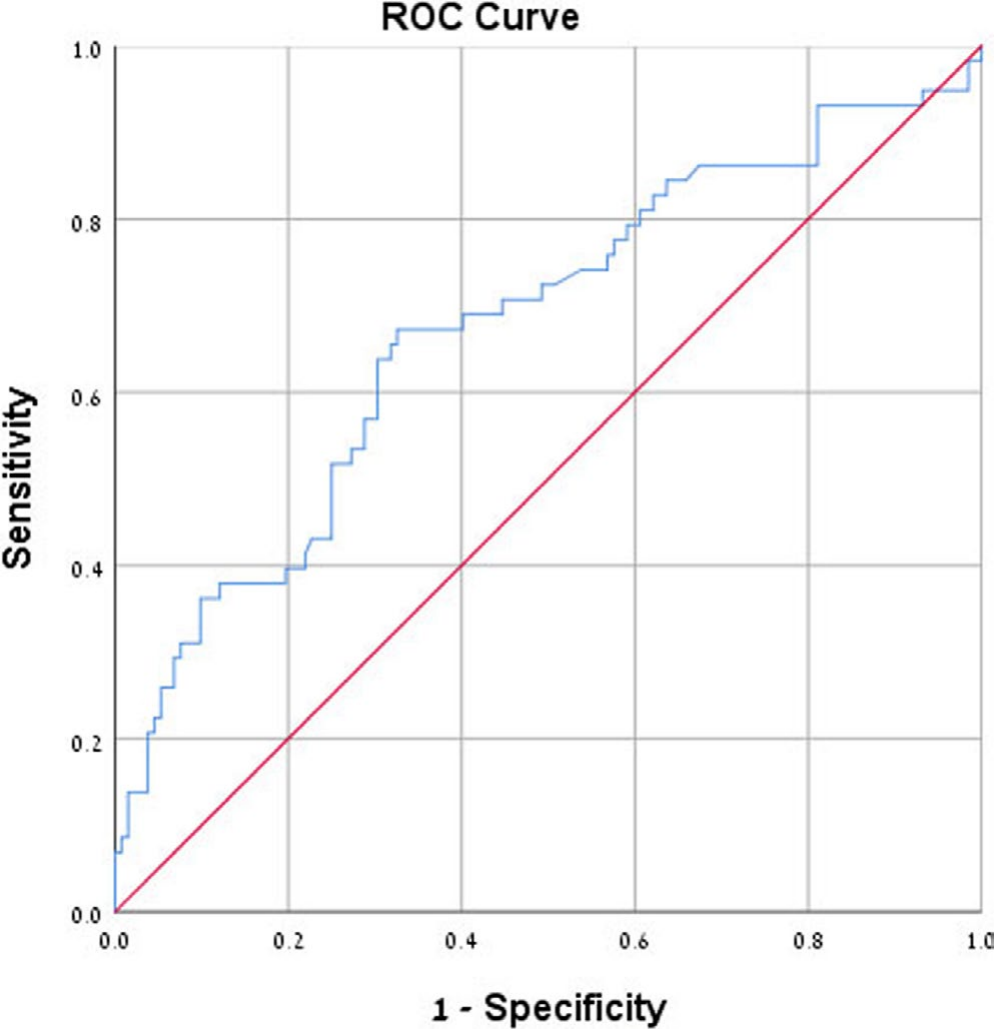
Receiver operating characteristic (ROC) curve analysis of neutrophil‐to‐lymphocyte ratio for microalbuminuria prediction. Area under curve is 0.675

**Figure 5:**
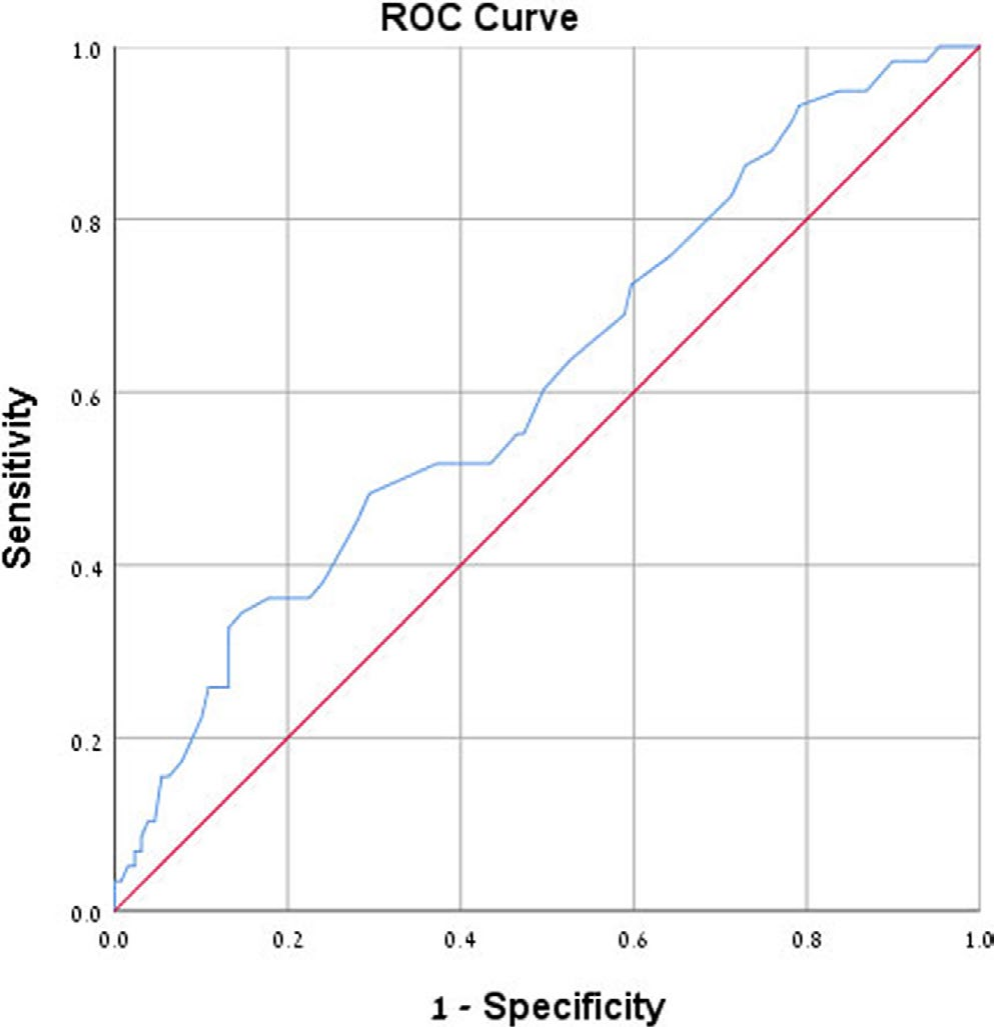
Receiver operating characteristic (ROC) curve analysis of red blood cell distribution width for microalbuminuria prediction. Area under curve is 0.614

Receiver operating characteristic curve analysis of NLR and RDW found an area under curve of 0.675 for NLR (confidence interval: 0.58‐0.76, *P* < .001) and 0.614 for RDW (confidence interval: 0.52‐0.70, *P* = .013) which suggests sufficient accuracy, presented graphically in Figures 4 and 5. Since NLR and RDW are newly proposed diagnostic parameters, we decided to examine them using the mean values of NLR and RDW found in Group C, diabetic patients with microalbuminuria, as cutoff points for predicting microalbuminuria. The data show that an NLR cutoff point of 2.54 has 39.7% sensitivity, 78.8% specificity, and 45% positive predictive value (PPV). An RDW cutoff point of 14.44 has 37.9% sensitivity, 76% specificity, and 41.5% PPV. Results of ROC curve analysis and selected cutoff points for NLR and RDW are presented in Table 4.

**Table 4:** Receiver operating characteristic (ROC) curve analysis for prediction of microalbuminuria using NLR and RDW cutoff values

Parameter	Cutoff	Sensitivity (%)	Specificity (%)	Positive predictive value (%)
NLR	2.54	39.7	78.8	45
RDW	14.55	37.9	76	41.5

Abbreviations: NLR, neutrophil‐to‐lymphocyte ratio; RDW, red blood cell distribution width.

## 4 DISCUSSION

The main purpose of this study was to evaluate the predictive value of hematological indices for microalbuminuria in type 2 diabetic patients. The sample was composed of patients with type 2 diabetes who were classified into three groups according to their levels of HbA1c and microalbuminuria. Levels of inflammatory markers were compared among the three groups of diabetic patients with and without microalbuminuria. The study findings indicated that high levels of NLR and RDW were found in diabetic patients with microalbuminuria compared to patients with controlled and uncontrolled diabetes without microalbuminuria. Moreover, NLR and RDW had a positive predictive value for microalbuminuria in diabetic patients.

In recent years, interest has grown in the study of inflammatory markers and their importance in diabetes and its complications. One study found that NLR is an independent predictor for microvascular complications in geriatric diabetic patients.^1^ Elevated NLR also found in otherwise healthy subjects that may be indicative of underlying impaired glucose metabolism, and moreover, NLR should be used as a marker of diabetic control level in addition to HbA1c in type 2 diabetic subjects.^9^ A retrospective Turkish study has also demonstrated a positive correlation between high levels of NLR, and HbA1c, serum creatinine, systolic blood pressure, and longer duration of diabetes, concluding that NLR is an efficient, cheaper, and readily available marker of inflammation, and it is known as an important predictor for the existence of microvascular complications in subjects with type 2 diabetes.^9^, ^10^

Another studies showed that RDW was significantly associated with HbA1c and that it may be regarded as a potential, innovative biomarker for improving risk assessment of developing diabetes**,** in addition RDW and RDW/MCV ratio were found associated with diabetic ketoacidosis (DKA) and valuable in predicting DKA 18, 19, 21. A significant positive relationship between microalbuminuria – a microvascular complication of diabetes – and MPV was also found in another study.^16^ One study also have showed that mean platelet volume to lymphocyte ratio (MPVLR) was an easily calculated and efficient index that can be considered a powerful and independent predictor of diabetic nephropathy in diabetic patients.^22^ In our study, we did not find any correlation between HbA1c and NLR or RDW. Moreover, we did not find any significant relationship between microalbuminuria and MPV.

A possible explanation for differences between the study groups may lie in the variables of age, years of diabetes, triglycerides/HDL (TG/HDL) ratio and serum creatinine level. Diabetes is a progressive disease and so it is likely that older patients will be included in the group of patients with microalbuminuria, as seen in the results of this study. The difference in TG/HDL ratio in our study is between the controlled‐diabetes group without microalbuminuria and both uncontrolled–diabetes groups with and without microalbuminuria. Perhaps the difference is due to metabolic syndrome and the fact that patients with complicated diabetes also have worsening metabolic syndrome. Further analysis found no correlation between higher TG/HDL ratio to elevated levels of inflammatory markers (NLR, RDW, MPV) and so it is not likely that increased TG/HDL ratio, was the cause for higher NLR or RDW in patients with microalbuminuria. There was no bias resulting in differences among the study groups regarding either blood pressure or LDL, which also reinforces the claim that metabolic syndrome is less likely to be the cause for the significant differences found between the groups. A higher level of serum creatinine was found in patients with microalbuminuria. An increase in serum creatinine is expected since microalbuminuria represents alteration in kidney function. Higher serum creatinine levels were also in correlation with increased NLR in this group. The professional literature seems to report no known direct correlation between an increase in serum creatinine and higher levels of inflammatory markers. Hence, we assume that the correlation found was comprised of independent elevations of serum creatinine and NLR in these patients. Diabetic nephropathy was the only cause for this increment of creatinine level. Since NLR was found to be significantly higher only in diabetic patients with microalbuminuria, compared to both controlled diabetes and uncontrolled diabetes, with no difference found between the latter two, we conclude that the elevations of NLR are primarily due to the presence of microalbuminuria, not the level of diabetes control. Even though the results showed a low sensitivity and positive predictive value for using NLR and RDW as markers of microalbuminuria, specificity for both parameters is above 70%. The use of these parameters could be beneficial for patient follow‐up, assuming that future studies prove its usefulness in the clinical practice.

## 5 CONCLUSIONS

This study showed that high levels of NLR were found in diabetic patients with microalbuminuria compared to controlled and uncontrolled patients without microalbuminuria. At the same time, we found high RDW values in patients with diabetes with microalbuminuria compared to diabetic patients without microalbuminuria. It seems that the primary cause of the elevations of NLR and RDW levels may be the presence of microalbuminuria and not the level of diabetes control. Moreover, NLR and RDW have a mild‐to‐moderate positive predictive value for microalbuminuria in type 2 diabetic patients. More prospective researches are still needed with a larger samples and follow‐up to reinforce their use and prove their effectiveness against urine testing in the future.

### 5.1 Limitations

Major limitations are the cross‐sectional design and lack of association with relevant outcome data. A larger‐scale research study is needed to further evaluate the application of NLR and RDW as prognostic factors for the prediction of microalbuminuria and other diabetic complications, as well as to formulate a systematic approach that would validate sufficient sensitivity and specificity and encompass the wide variety of diabetic and other medical complications. Other limitation is the lack of association with relevant outcome data.

## AUTHOR CONTRIBUTIONS

RF and TA contributed to the concept of the study. TA collected the data and performed the statistical analysis. TA, RF, and WN wrote the manuscript. TA, RF, RKF, AB, and WN revised the manuscript.
